# High Levels of Asymmetric Dimethylarginine Are Strongly Associated with Low HDL in Patients with Acute Myocardial Infarction

**DOI:** 10.1371/journal.pone.0064796

**Published:** 2013-06-06

**Authors:** Julie Lorin, Jean-Claude Guilland, Claudia Korandji, Claude Touzery, Florence Bichat, Aline Chagnon, Yves Cottin, Luc Rochette, Catherine Vergely, Marianne Zeller

**Affiliations:** 1 INSERM UMR866– Laboratory of Cardiometabolic Physiopathology and Pharmacology, University of Burgundy, Dijon, France; 2 Cardiology Department, University Hospital, Dijon, France; Albany Medical College, United States of America

## Abstract

**Objectives:**

Low levels of high-density lipoprotein (HDL) cholesterol are associated with an increased risk of acute myocardial infarction possibly through impaired endothelial atheroprotection and decreased nitric oxide (NO) bioavailability. Asymmetric dimethylarginine (ADMA) mediates endothelial function by inhibiting nitric oxide synthase activity. In patients with acute myocardial infarction, we investigated the relationship between serum levels of HDL and ADMA.

**Approach and Results:**

Blood samples from 612 consecutive patients hospitalized for acute MI <24 hours after symptom onset were taken on admission. Serum levels of ADMA, its stereoisomer, symmetric dimethylarginine (SDMA) and L-arginine were determined using high-performance liquid chromatography. Patients with low HDL (<40 mg/dL for men and <50 mg/dL for women) were compared with patients with higher HDL. Most patients (59%) had low HDL levels. Median ADMA levels were markedly higher in the low HDL group (0.69 vs. 0.50 µmole/L, p<0.001). In contrast, SDMA and L-arginine levels were similar for the two groups (p = 0.120 and p = 0.064). Notably, ADMA, but not SDMA or L-arginine, was inversely correlated with HDL (r = −0.311, p<0.001). In stratified analysis, this relationship was only found for low HDL levels (r = −0.265, p<0.001), but not when HDL levels were higher (r = −0.077, p = 0.225). By multivariate logistic regression analysis, ADMA level was strongly associated with low HDL levels (OR(95%CI):6.06(3.48–10.53), p<0.001), beyond traditional confounding factors.

**Conclusions:**

Our large population-based study showed for the first time a strong inverse relationship between HDL and ADMA in myocardial infarction patients, suggesting a functional interaction between HDL and endothelium, beyond metabolic conditions associated with low HDL levels.

## Introduction

Reduced endothelial nitric oxide (NO) bioavailability contributes to the development and progression of atherosclerosis, leading to coronary artery disease (CAD). Asymmetric dimethylarginine (ADMA), an endogenous competitive inhibitor of all isoforms of nitric oxide synthases (NOS), may compete with L-arginine as the substrate for the enzyme or inhibit NOS phosphorylation and thus decrease NO bioavailability [1], [2]. ADMA and its stereoisomer, symmetric dimethylarginine (SDMA) are endogenously produced by the methylation of arginine residues from nuclear proteins and are released after proteolysis. Unlike ADMA, which is a primary factor in the control of NOS activity, SDMA has insignificant inhibitory effects on the enzyme. High ADMA concentrations have been associated with most cardiovascular risk factors, including dyslipidemia [3], [4]. Moreover, several lines of evidence have demonstrated that elevated ADMA levels are associated with endothelial dysfunction in healthy subjects and in patients with CAD or hypercholesterolemia^,^ [3], [5].

Epidemiological studies and randomized clinical trials have showed that low plasma levels of high-density lipoprotein (HDL) are associated with an increased risk of cardiovascular events in CAD patients, suggesting that HDL-C (HDL cholesterol) has cardioprotective effects [6]. This strong relationship has stimulated interest in determining mechanisms and optimal management of low levels of HDL-C. Beyond reverse cholesterol transport, the molecular mechanisms underlying the anti-atherogenic properties of HDL-C include antioxidant, anti-inflammatory and vasculo-protective effects [7]. Moreover, experimental studies have identified various direct endothelial protective effects of HDL-C, including the stimulation of endothelial NO production [8]. HDL-C restores endothelial function by increasing NO bioavailability [9]. Decreased HDL-C levels are frequently associated with the defective functionality of HDL-C particles, which have abnormal metabolism and chemical composition, reflecting in part their attenuated cholesterol efflux capacity but also their decreased anti-inflammatory and antioxidative activities [10]. However, there is little information on the association between ADMA and HDL-C, limited to an in vitro study using human umbilical vein endothelial cells (HUVECs) [11]. From a large prospective population of MI patients, we hypothesized that elevated circulating levels of ADMA, as a surrogate marker of endothelial health, could be associated with low HDL-C levels.

## Patients and Methods

### Study Subjects

All the consecutive patients aged >18 y and hospitalized <24 hours after symptom onset for acute MI in the coronary care unit of Dijon University Hospital between 1^st^ January 2011 and 30^th^ June 2012 were included. Patients with relevant co-morbidities (infection, autoimmune disorders and cancers) were excluded from the study. MI was defined by an increase in serum troponin I (2X> upper limit of the hospital normal (ULN) range) associated with symptoms of ischemia and/or characteristic ECG signs (ST-segment-T wave changes, left bundle branch block, or development of pathological Q waves) [12]. STEMI was defined by new ST-segment elevation >1 mm or left bundle branch block on the qualifying ECG.

The study was approved by the Consultative Committee of Protection of Persons in Biomedical Research of Burgundy and conducted in accordance with Declaration of Helsinki. All subjects gave their written consent to participate in the study.

### Group Definition

Patients were analyzed according to their HDL-C levels. Low HDL-C was defined by <40 mg/dL in men and <50 mg/dL in women [13].

### Data Collection

Data on demographics, risk factors [history of hypertension, diabetes, hyperlipidemia, body mass index (BMI)], chronic treatments and prior MI were prospectively collected. Echocardiography was performed at 2±1 days and the Simpson method was used to assess left ventricular ejection fraction (LVEF).

### Biological Data

Blood samples were drawn on admission (Median time from symptom onset to blood sampling: 16(8–30) hours). Homocysteine concentrations were determined using a chemiluminescence method on an Immulite 2000 analyzer (Diagnostic Products Corporation, Los Angeles, USA). C-reactive protein (CRP), total cholesterol (TC), high-density lipoprotein cholesterol (HDL-C) and triglyceride (TG) concentrations were measured on a Dimension analyzer (Dade Behring, Newark, NE). The level of low-density lipoprotein cholesterol (LDL-C) was calculated using the Friedewald formula [14]. Plasma glucose concentrations (enzymatic method (glucose oxidase)) and creatinine levels were measured on a Vitros 950 analyzer (Ortho Clinical Diagnostics, Rochester, NY) and creatinine clearance was calculated with the Cockcroft formula. Glycated hemoglobin A1c (HbA1c) was measured with ion exchange HPLC (Bio-Rad Laboratories, Richmond, CA).

### Dimethylarginines and L-arginine Analysis

Samples were allowed to clot at room temperature for 30 minutes and centrifuged at 2500 rpm for 10 minutes at 4°C. The serum was kept frozen at −80°C until analysis. L-arginine, ADMA, and SDMA, were measured by high performance liquid chromatography (HPLC) [15]. Before the analysis, serum was added with N-monomethyl L-arginine (NMMA) as the internal standard and phosphate–buffered saline (PBS). This mixture was extracted on solid-phase extraction (SPE) cartridges (Phenomenex Strata X-C, Torrance, CA, USA). The cartridges were first conditioned with elution buffer (10/0.5/40/50; NH_3_ concentrated/1 M NaOH/bidistilled water/CH_3_OH; v/v/v/v) followed by bidistilled water before being loaded with the diluted sample. The SPE cartridge was consecutively washed with HCl (100 mmol/L) and methanol (1∶1; v:v).ADMA and SDMA were eluted with elution buffer. All conditioning, washing and elution steps were achieved by vacuum suction. The eluate was dried under nitrogen, derivatized with ortho-phthaldialdehyde (OPA) reagent (1∶1; v:v) and injected into the HPLC system, with a fluorescent detector Finingan Surveyor (Thermo Fisher (λexc:340 nm, λem:455 nm) and Chromolith® RP-18E column (100×4.6 mm) including a guard cartridge (10×4.6 mm) supplied by Merck (Darmstadt, Germany). Chromatographic separation, at room temperature, was performed isocratically at 100% mobile phase A, with 25 mmol/L phosphate buffer (pH 6.8) containing 6.5% CH_3_CN, at a flow rate of 1.1 mL/min. After SDMA elution, mobile phase was switched to 100% mobile phase B, with ultrapure water:CH_3_CN (50∶50, v:v), and the flow rate was increased to 3.0 mL/min to elute strongly retained compounds. Assays were performed in duplicate.

The detection limits were 0.05 and 1.19 µmol/L and interday variabilities were 5.7 and 4.6% for ADMA and L-arginine, respectively.

### Statistical Analysis

All the analyses have been made on anonymous de-identified data that were available to others. Data are presented as median (interquartile range [IQR]), or proportion (n(%)). For continuous variables, normality was checked by the Kolmogorov-Smirnov test. The Student t test or Mann-Whitney test was used to test for continuous data in the two groups. Categorical variables were compared by the chi-square test or Fisher’s exact test. Spearman’s correlation was applied to test for associations between dimethylarginines, L-arginine and biological variables. Mann-Whitney test was used to test L-arginine and dimethylarginines concentrations in patients according to their HDL levels.

In order to further investigate whether the relationship between HDL-C and ADMA was influenced by metabolic or pharmacological conditions, stratified correlation analysis was performed on subgroups of patients based on median LDL or CRP values (inframedian or supramedian), on the chronic use of statin or fibrate therapy (with or without treatment) and on levels of HDL-C (low or high HDL-C). Factors associated with low HDL-C were studied by backward logistic regression analysis. Variables entered into the multivariate model were chosen according to their relationship (cut-off at 10%) in univariate analysis (i.e. age, BMI, diabetes, prior MI, fibrate, CRP, HbA1c, triglycerides, ADMA, SDMA, L-arginine and smoking), with an exclusion cut-off at 1%. All the analyses were performed using the SPSS 13.0 software package (IBM Inc, USA).

## Results

### Baseline Characteristics

**Figure 1:**
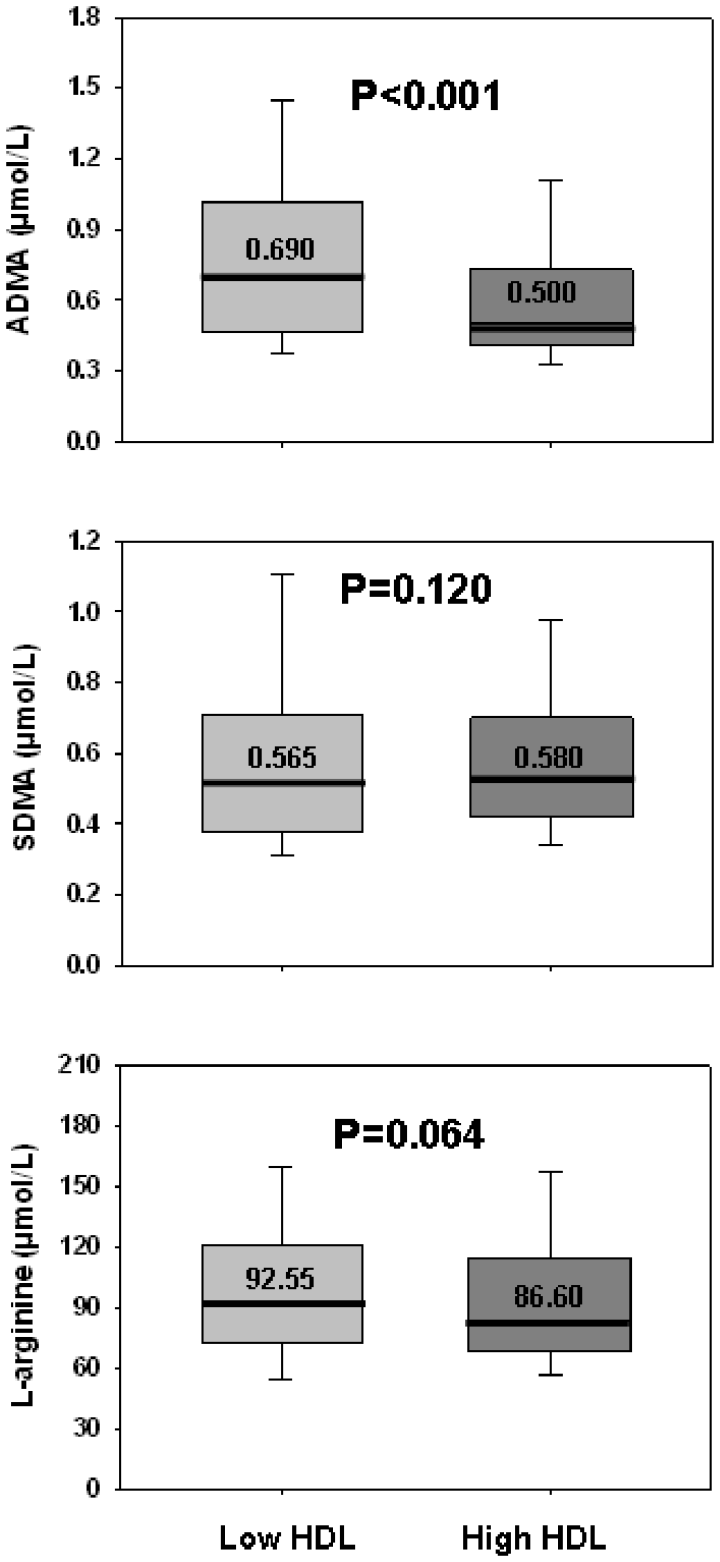
L-arginine and dimethylarginines concentrations in patients according to their HDL levels. The horizontal line is the median, box is the IQR (interquartile range) and whiskers are the 95% range. P values are from Mann-Whitney tests.

Patients’ characteristics are shown in Table 1. Most patients (362/612 (59%)) had low HDL-C levels. Patients with low HDL-C were younger, more frequently smokers, with prior MI, had a higher body mass index, and showed a trend towards a greater rate of diabetes than patients with high HDL-C. Clinical data and current medications were similar for the two groups, except for fibrate therapy, which was more frequent in the low HDL-C group. Biological data are shown in Table 2. Higher CRP and triglycerides and a trend toward higher HbA1c levels were found in patients with low HDL-C. No difference was observed between the two groups for LDL-C, glucose, creatinine clearance and homocysteine. Interestingly, median ADMA concentrations were markedly higher (∼ 40%, i.e. 0.69 vs. 0.50 µmole/L, p<0.001) in patients with low HDL-C than in those with higher HDL-C levels (Figure 1). In contrast, there was no or only weak difference between the two groups for SDMA and L-arginine levels (p = 0.120 and p = 0.064, respectively).

**Table 1:** Patients’ characteristics (n(%) or median (IQR)).

	High HDL	Low HDL	p
	N = 250	N = 362	
**Risk factors**			
Age, *years*	68(57–79)	65(52–77)	0.007
Female	60(24)	99(27)	0.353
BMI, *kg/m^2^*	26(24–29)	27(25–30)	0.002
Hypertension	136(55)	200(55)	0.934
Hypercholesterolemia	111(44)	155(43)	0.658
Family history of CAD	65(26)	100(28)	0.521
Diabetes	45(18)	86(24)	0.085
Smoking	62(25)	130(36)	0.002
Prior myocardial infarction	27(11)	65(18)	0.015
**Clinical data**			
HR, *beats/min*	77(67–90)	76(66–88)	0.542
SBP, *mmHg*	140(121–163)	137(120–160)	0.308
DBP, *mmHg*	80(70–96)	80(69–90)	0.093
STEMI	147(59)	188(52)	0.093
LVEF, *%*	55(45–60)	55(45–60)	0.670
**Current medications**			
Statin	66(26)	92(25)	0.784
Fibrate	7(3)	23(6)	0.045
ACE inhibitor	42(17)	74(20)	0.258
Aspirin	56(22)	78(21)	0.802

Low HDL was defined as <40 mg/dL in men and <50 mg/dL in women.

ACE: Angiotensin converting enzyme; BMI: body mass index; CAD: Coronary artery disease; DBP: Diastolic blood pressure; HR: heart rate; LVEF: Left ventricular ejection fraction; SBP: Systolic blood pressure; STEMI: ST segment elevation myocardial infarction.

**Table 2:** Biological data (Median (IQR)).

	High HDL	Low HDL	p
	N = 250	N = 362	
CRP, *mg/L*	4.0(3.0–10.7)	6.5(3.0–18.0)	<0.001
Homocysteine, *µmol/L*	13(10–17	12(10–17	0.239
Creatinine clearance, *ml/min*	75(54–92)	80(52–107)	0.311
Glucose, *mmol/L*	6.84(5.72–8.37)	6.93(5.77–9.32)	0.211
HbA1c, *%*	5.8(5.5–6.3)	5.9(5.6–6.7)	0.082
HDL-cholest, *mg/dL*	54(47–64)	33(27–38)	<0.001
LDL-cholest, *mg/dL*	121(94–152)	125(94–153)	0.390
Total-cholest, *mg/dL*	199(168–227)	191(158–224)	0.023
Triglycerides, *mg/dL*	93(68–135)	138(95–199)	<0.001
ADMA, *µmol/L*	0.50(0.41–0.73)	0.69(0.47–1.02)	<0.001
SDMA, *µmol/L*	0.52(0.42–0.70)	0.51(0.38–0.71)	0.120
L-arginine, *µmol/L*	83.9(68.7–114.4)	91.1(72.5–120.9)	0.064
L-arg/ADMA ratio	163(118–225)	132(92–199)	<0.001

Low HDL was defined as HDL <40 mg/dL in men and <50 mg/dL in women.

ADMA: Asymmetric dimethylarginine; CRP: C-reactive protein; HbA1c: glycated hemoglobin; HDL-C: high-density lipoprotein; LDL-C: low-density lipoprotein; SDMA: Symmetric dimethylarginine.

### ADMA and HDL-C

**Figure 2:**
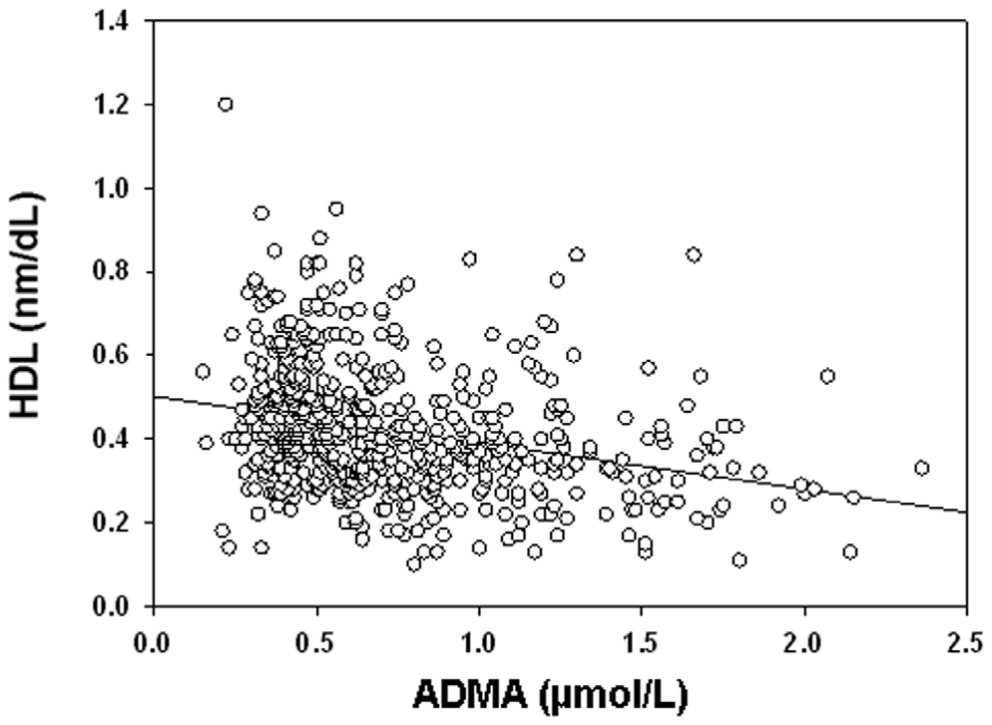
Correlations between ADMA and HDL circulating levels (r = −0.311, p<0.001).

Spearman correlation analyses for ADMA, SDMA, L-arginine and biological parameters are reported in Table 3. ADMA was positively related to SDMA and L-arginine, with a trend towards a negative relationship with homocysteine (p = 0.067). Moreover, ADMA showed a strong negative correlation with HDL-C (r = −0.311, p<0.001) (Figure 2). SDMA had only a weak and positive association with HDL-C (p = 0.040), but a strong correlation with both LDL and homocysteine (p<0.001). L-arginine was associated positively with LDL and triglyceride levels, but negatively with HDL-C (p = 0.006). Subgroup correlation analysis showed that whatever the LDL and CRP levels (i.e. stratified by median values) and treatments (stratified by statin or fibrate use), the significant relationship between ADMA and HDL-C was maintained in all subgroups (p<0.001). In contrast, when patients were classified according to HDL-C levels (stratified by low/high HDL-C) (Table 4), only patients with low HDL-C levels showed a strong relationship between HDL-C and ADMA (r = −0.265, p<0.001). In contrast, there was no significant correlation between these two parameters in patients with higher HDL-C (p = 0.225).

**Table 3:** Correlations between dimethylarginines, L-arginine and biological data (N = 612).

	ADMA		SDMA		L-arginine	
	r	p	r	p	r	p
ADMA	–	–	0.248	<0.001	0.260	<0.001
SDMA	0.248	<0.001	–	–	0.057	0.162
L-arginine	0.260	<0.001	0.057	0.162	–	–
HDL-Cholest	−0.311	<0.001	0.083	0.040	−0.111	0.006
LDL-Cholest	0.070	0.088	−0.193	<0.001	0.169	<0.001
Total-Cholest	−0.041	0.309	−0.163	<0.001	0.144	<0.001
Triglycerides	−0.009	0.825	−0.074	0.068	0.106	0.009
Creatinine clearance	−0.078	0.061	−0.448	<0.001	0.107	0.010
Homocysteine	−0.077	0.067	0.386	<0.001	−0.042	0.321
HbA1c	−0.072	0.087	0.029	0.495	−0.038	0.368
CRP	0.051	0.212	0.145	<0.001	−0.025	0.546
Glucose	0.014	0.737	0.009	0.821	−0.060	0.142

ADMA: Asymmetric dimethylarginine; CRP: C-reactive protein; HbA1c: glycated hemoglobin; HDL-C: high-density lipoprotein; LDL-C: low-density lipoprotein; SDMA: Symmetric dimethylarginine.

**Table 4:** Correlations between dimethylarginines, and Larginine in the high and low HDL groups.

	ADMA			
	HighHDL		LowHDL	
	N = 250		N = 362	
	r	p	r	p
HDL-Cholest	−0.077	0.225	−0.265	<0.001
SDMA	0.319	<0.001	0.253	<0.001
L-arginine	0.171	0.007	0.311	<0.001

Low HDL was defined as <40 mg/dL in men and <50 mg/dL in women.

ADMA: Asymmetric dimethylarginine; HDL-C: high-density lipoprotein; SDMA: Symmetric dimethylarginine.

**Figure 3:**
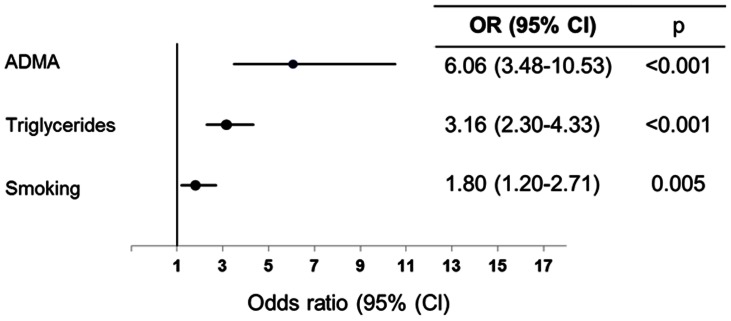
Multivariate backward logistic regression analysis with low HDL levels as the dependent variable.

Backward logistic regression analysis with low HDL-C as a dependent variable showed that the level of ADMA was strongly associated with the HDL-C levels (OR(95%CI): 6.06(3.48–10.53), p<0.001), beyond confounding factors such as triglycerides (OR(95%CI):3.16(2.30–4.33), p<0.001) and smoking (OR(95%CI): 1.80(1.20–2.71),p = 0.005) (Figure 3). SDMA was not independently associated with HDL-C in the final multivariate model. The Quality Index validated the ability of the model to correctly predict HDL-C (−2LL = 664.72, p (Hosmer-Lemeshow) = 0.540, with more than 75% of correct classifications in the at-risk group).

## Discussion

In recent years, it has been established that HDL-C may exert atheroprotective effects through its beneficial effect on endothelial cells. However, only few data are available in humans on the relationship between HDL-C levels and their association with ADMA, as a surrogate marker of endothelial health. Our study is the first large prospective study to show that patients with low HDL-C levels were characterized by markedly high ADMA levels, whatever the levels of lipids and inflammation markers. Given that this relationship between ADMA and HDL-C levels was only found in patients with low HDL-C levels, our work also lends further support to the concept of the functional heterogeneity of HDL-C in such high-risk patients.

### ADMA and Endothelial Dysfunction

In MI patients, circulating ADMA has been independently associated with clinical outcomes probably through its deleterious impact on vascular homeostasis [16], [17]. The role of ADMA as an endogenous competitive inhibitor of eNOS is now well established [18], [19]. High plasma ADMA concentrations are associated with endothelial dysfunction in patients with CV risk factors or CAD, while this association is weak in healthy individuals[20]–[23]. Endothelial dysfunction is primarily characterized by impaired NO-induced vasodilation, and associated with traditional CV risk factors such as dyslipidemia, arterial hypertension and metabolic syndrome [3], [5], [24]. More precisely, in the vascular endothelium of CAD patients, high serum ADMA levels are associated with impaired vascular function reflected by increased vascular O_2_^•−^ production and reduced NO bioavailability, due to accelerated eNOS uncoupling [25].

### Low HDL-C and Dysfunctional HDL-C

Our study, like other recent studies [26], showed that most patients with acute MI had low HDL-C levels. Low HDL-C is an independent risk factor for CAD, whatever the level of LDL-C. However, the effects of therapies to raise HDL-C, such as cholesterylester transfer protein (CETP) inhibitors, are controversial, partly because of the complex interaction of the lipoprotein particle with the enzyme, which depends on the metabolic context [27]. It is becoming increasingly evident that HDL-C particles not only promote reverse cholesterol transport from the periphery (mainly macrophages) to the liver but also exert pleiotropic effects, in particular on inflammation and vascular homeostasis. However, few studies have been carried out on the association between low HDL-C levels and markers of endothelial dysfunction.

In our study, patients with low HDL-C were more likely to have metabolic risk factors such as diabetes, obesity and high triglycerides, characteristics of the atherogenic dyslipidemia phenotype [28], [29]. Moreover, active smokers have lower levels of HDL-C and HDL particles, and both increase with smoking cessation [30], [31]. In our study population, whether low HDL could be associated with qualitative modifications in HDL-C remains speculative. However, consistent data have showed that reductions in HDL-C levels frequently coincide with HDL-C functional impairment and alterations in HDL-C metabolism and structure. In common metabolic diseases, such as type 2 diabetes and metabolic syndrome, low circulating levels of HDL-C are associated with the defective functionality of small HDL particles, which also have an abnormal structure and composition.

Moreover, in atherogenic low HDL-C dyslipidemia, circulating levels of large, cholesterol-rich HDL2 decrease in parallel with HDL-C [32], [33]. The biological activities of atheroprotective HDL-C (i.e. HDL3) are also often compromised when levels of HDL-C are low. Specifically, in CAD, alterations in HDL3 composition, which include decreases in cholesteryl esters, apoA-I, and paraoxonase 1 (PON1) together with increases in triglyceride and serum amyloid A (SAA) and covalent modifications of HDL-C by oxidation and/or glycation, cause a decrease in cellular cholesterol capacity as well as decreases in the antioxidative and anti-inflammatory activities of these particles. In addition, acute phase protein SAA, which is bound by HDL-C, particularly HDL_3_, has recently been shown to promote endothelial dysfunction and reduce NO bioavailability by stimulation of O_2_ production [34], [35]. The ability of HDL to stimulate the eNOS activating pathway was impaired in CAD patients, in part due to reduced PON1-HDL-associated activity [36]. Moreover, low HDL-C has also recently been reported as an important determinant of lipid peroxidation, irrespective of risk factors and inflammatory and/or metabolic biomarkers [37].

### ADMA, Endothelial Dysfunction and HDL-C

Recent studies on endothelial cell cultures or isolated vessels showed that HDL-C can exert direct biological effects on endothelial cells, via stimulation of eNOS mediated NO production, increased eNOS expression and NO-mediated vasodilation [7]. In humans, consistent data also show that under certain favorable conditions, HDL-C could exert beneficial effects on endothelium-dependent vascular reactivity. In CAD patients, HDL-C was significantly related to the Flow Mediated Dilation (FMD) of brachial arteries (beta = 0.295, P = 0.018) [38].

The underlying mechanisms of the effects of HDL-C on eNOS stimulation remain unclear. Early studies have suggested that HDL-C can prevent the detrimental effects of LDL oxidation on eNOS. In human umbilical vein endothelial cell cultures, exposure to increasing HDL-C concentrations protected against reduced NO production induced by ox-LDL, with concomitant decreases in ADMA levels [11]. However, one emerging hypothesis, based on experimental findings, suggests an effect on apoA-I and ABC transporters (ABCG1), eNOS phosphorylation-induced activation of PI3K/Akt, MAP kinase pathways and Scavenger receptor B type 1 (SRB1), or interaction of eNOS with caveolin-1 [33], [39]. On the other hand, HDL-associated lysophospholipid, i.e. shingosine-1-phosphate (S1P), binds to the lysophospholipid receptor S1P3, thus stimulating the Akt pathway and thereby eNOS phosphorylation at Ser177. HDL-C also contains active PON1 which suppresses the formation of malondialdehyde (MDA), and thereby exerts beneficial effects on the endothelium through blunting lectin-type oxidized LDL receptor 1 (LOX-1)–induced inhibition of eNOS phosphorylation.

Our study provides important additional data that suggest a specific role of HDL-C in ADMA-related endothelial dysfunction. Our data are in agreement with the findings of recent studies to assess the impact of strategies to raise HDL-C on endothelial function. In patients with type II hyperlipidemia or CAD, a 16 to 26% increase in HDL-C plasma levels was achieved by a CETP inhibitor or niacin, and was associated with improved endothelial function, but only in the subgroup of patients with low baseline HDL-C levels [40], [41]. When HDL-C level achieved is sufficient (i.e. >40 mg/dL in men and >50 mg/dL in women), corresponding to physiological particle sizes and functionality, the lack of any significant interaction between ADMA and HDL-C strongly suggests that other pathophysiological pathways, including Ca^2+^-activated calmodulin, Hsp90, shear stress, oestrogen and vascular endothelial growth factor (VEGF), are involved in regulating eNOS activity [42]. Moreover, as SDMA, which is not a competitive inhibitor of NO synthase, failed to show any independent relationship with the lipoprotein, the link between ADMA and HDL-C levels may be due to the modulation of eNOS activity. The recent disappointing results from the dal-VESSEL randomized clinical trial, showing a lack of improvement in brachial artery reactivity, as a surrogate of endothelial function, and inflammatory markers after 36 weeks of treatment with dalcetrapib, also add fuel to this hypothesis of the functional heterogeneity of HDL particles in CAD patients [43].

### Study Limitations

As suggested by earlier studies, lipoprotein-related parameters may vary over the few days following MI. However, previous reports have indicated that lipid changes in response to MI-related inflammation are only minor during the first 24 h of MI [44], [45]. Thus, in the present study, it can be assumed that measurements of HDL-C, LDL and triglycerides at the early phase of acute MI reflect accurately and in a retrospective manner the lipoprotein profile before the MI as well as during the acute phase. The main strength of this study is the use of large prospective population-based registry, with adjustments for a wide range of possible confounding factors. These elements limit the risk of any bias in our conclusions. This work, however, suffers from the usual limitations of observational, non-randomized studies and therefore determines correlations rather than causal relationships.

### Conclusions

The adverse effects or the lack of clinical benefit of strategies to raise HDL-C suggest that better understanding of the link between HDL-C and markers of vascular homeostasis is urgently required, as are efficient methods to measure HDL functionality [46]. ADMA is now emerging as a relevant clinical tool, and a useful biomarker of endothelial dysfunction. Some authors have proposed ADMA as an in vivo assessment of endothelial health and even suggested that it could be used to monitor the vascular effects of statin therapy in a clinical setting [47]. This study points out how the implementation of such surrogate markers in clinical trials could give a measure of vascular effects of the lipoprotein and thus may allow for the shortening of clinical trials, which could in turn help to reduce the cost of drug development in the future. Our large population-based study further supports the hypothesis of the functional heterogeneity of HDL particles in CAD patients, and strongly suggests that the biological activity of on-treatment HDL-C, in particular regarding endothelial health, in addition to its plasma levels, needs to be taken into account for the development of targeted strategies to raise HDL levels or to treat HDL dysfunction.
